# Magnetic-Particle-Sensing Based Diagnostic Protocols and Applications

**DOI:** 10.3390/s150612983

**Published:** 2015-06-04

**Authors:** Tsukasa Takamura, Pil Ju Ko, Jaiyam Sharma, Ryoji Yukino, Shunji Ishizawa, Adarsh Sandhu

**Affiliations:** 1 Electronics-Inspired Interdisciplinary Research Institute (EIIRIS), Toyohashi University of Technology, 1-1 Hibarigaoka, Tempaku, Toyohashi, Aichi 441-8580, Japan; E-Mail: sandhu@uec.ac.jp; 2 Research Promotion Center, The University of Electro-Communications, 1-5-1 Chofugaoka Chofu, Tokyo 182-8585, Japan; E-Mail: pjko77@uec.ac.jp; 3 Department of Electrical and Electronic Information Engineering, Toyohashi University of Technology, 1-1 Hibarigaoka, Tempaku, Toyohashi, Aichi 441-8580, Japan; E-Mails: jaiyam@eiiris.tut.ac.jp (J.S.); yukino@eiiris.tut.ac.jp (R.Y.); ishizawa@eiiris.tut.ac.jp (S.I.); 4 Department of Engineering Science, University of Electro-Communications, 1-5-1 Chofugaoka, Chofu, Tokyo 182-8585, Japan

**Keywords:** magnetic particles, medical diagnosis, magnetic sensor, self-assembly, porous silicon

## Abstract

Magnetic particle-labeled biomaterial detection has attracted much attention in recent years for a number of reasons; easy manipulation by external magnetic fields, easy functionalization of the surface, and large surface-to-volume ratio, to name but a few. In this review, we report on our recent investigations into the detection of nano-sized magnetic particles. First, the detection by Hall magnetic sensor with lock-in amplifier and alternative magnetic field is summarized. Then, our approach to detect sub-200 nm diameter target magnetic particles via relatively large micoro-sized “columnar particles” by optical microscopy is described. Subsequently, we summarize magnetic particle detection based on optical techniques; one method is based on the scattering of the magnetically-assembled nano-sized magnetic bead chain in rotating magnetic fields and the other one is based on the reflection of magnetic target particles and porous silicon. Finally, we report recent works with reference to more familiar industrial products (such as smartphone-based medical diagnosis systems and magnetic removal of unspecific-binded nano-sized particles, or “magnetic washing”).

## 1. Introduction

In recent decades, magnetic particles or magnetic beads have attracted huge attention due to their specific abilities. Magnetic particles hold several advantages, not least their large surface-to-volume ratio, their ability to be manipulated by magnetic field gradient application, and the fact they are harmless to the human body [1]. The size of magnetic particles is also controllable within the range of a few nano-meters to a few micron meters depending on the desired purpose. Owing to such advantages, magnetic particles are being used in magnetic resonance imaging (MRI), treatment of hyperthermia, drug delivery, cell and biological material manipulation and separation, and several other fields in medical applications [2]. Here, we summarized our recent works related to magnetic detection by magnetic sensors [3,4,5,6,7]. We also describe novel techniques utilizing columnar magnetic particles to detect magnetic nano-sized particles which are difficult to observe by typical optical microscope and difficult to detect by magnetic sensor due to the sensor’s intrinsic noise [8,9,10,11]. Subsequently, two of our optical detection methods are presented [12,13]. Finally, our recent works connected with industrial products are introduced [14]. 

## 2. Magnetic Particle Detection by Hall Magnetic Sensor 

**Figure 1:**
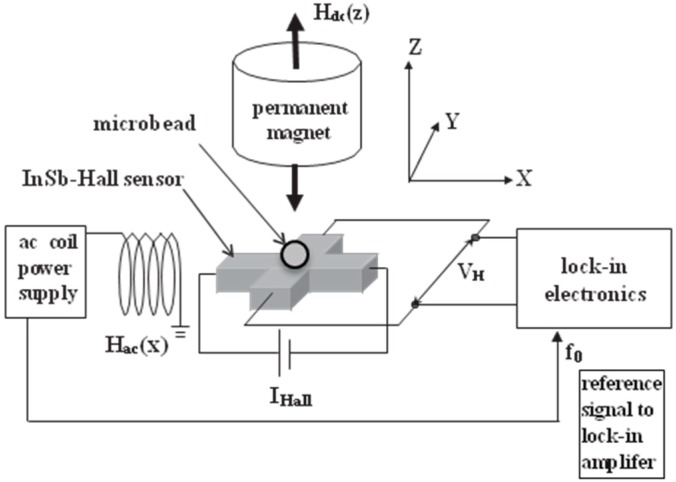
The system for detecting magnetic particles with a Hall sensor. Adapted from [3].

Our first work relates to the detection of magnetic particle by InSb Hall sensors [3].The method we adopted was inspired by our research into scanning Hall probe microscopy [4,5,6]. Figure 1 shows the system we used to detect magnetic particle with Hall sensors. It consists of a coil for generating alternative magnetic field in x direction, a magnet for applying perpendicular magnetic field to the sensor, and a Hall sensor connected to the lock-in amplifier to monitor the output voltage of the Hall sensor.

**Figure 2:**
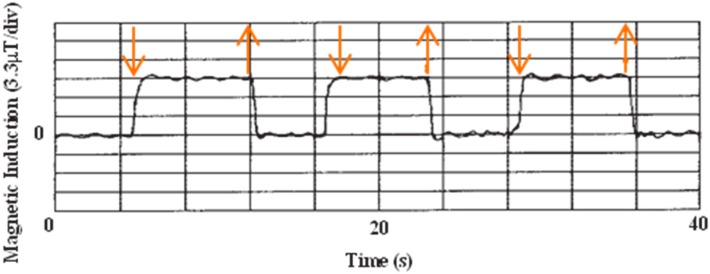
Real time variation of the magnetic induction on the InSb micro-Hall sensor due to the presence of a single micro-bead as measured by monitoring changes in Hall voltage after the application (downward arrow) and removal (upward arrow) of the 320 Oe by magnet. The ac in-plane field was 44 Oe at 670 Hz and lock-in time constant was 100 ms. Adapted from [3].

The particle we used was a superparamagnetic bead (SPB) which has no residual magnetization. The difference of the vertical component magnetic particles can be detected by monitoring at twice the frequency of the applied alternating in-plane field. Without a perpendicular magnetic field, the magnetic particle on the sensor surface is just magnetized in the x direction by an alternative magnetic field generated from the coil. Because Hall sensors can only detect the magnetic field perpendicular to the sensor, the sensor output is almost zero in such conditions. When the magnet was close enough to the sensor to apply a vertical magnetic field and the field is sufficient to saturate the magnetization of the magnetic particle, the vertical component of the particle’s magnetization is changed periodically with twice the frequency of the x direction alternative magnetic field. Figure 2 shows the results of magnetic particles on the Hall sensor using our system. The particle was a 2.8 μm diameter magnetic bead (Dynabeads M-270). The signal was clearly obtained when applying a perpendicular magnetic field.

The signal shown in Figure 2 was from a particle placed just on the sensor by a micromanipulator, and there was no connection between the particle and sensor surface. We also detected DNA molecules labeled with 200 nm diameter magnetic beads by the same detection method [7] and showed the potential for real medical diagnosis.

## 3. The Detection via Columnar Particle Magnetically Captured by Nano-Sized Target Particles

### 3.1. The Concept and Detection of Nano-Sized Target Particles via Columnar Particles 

**Figure 3:**
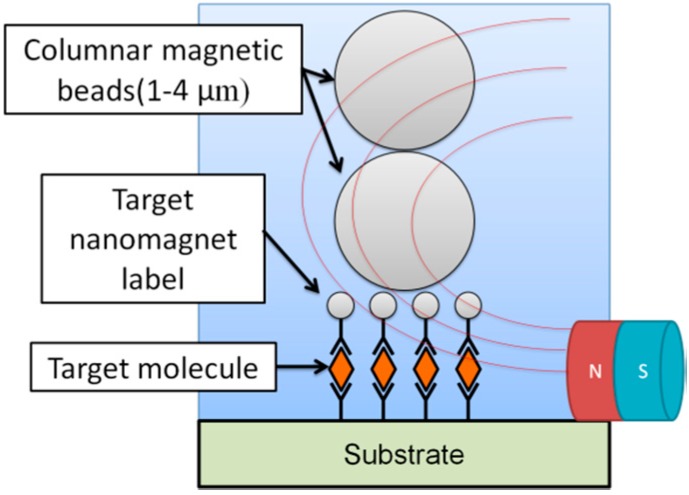
Our concept for a simple method to detect nano-sized magnetic particles via magnetically captured micro-sized magnetic particles using an optical microscope. Adapted from [8].

As discussed above, we developed a Hall sensor-based magnetic particle detection system and successfully detected micro- and nano-sized particles on the sensor surface. For more practical applications, the detection of nano-sized magnetic particles is needed to improve quantification and prevent steric hindrance. However, the detection of nano-sized magnetic particles by typical magnetic sensors still faces difficulties due to intrinsic noise, particularly in the case of detecting the small numbers of magnetic nano-sized particles. To overcome this difficulty, we demonstrated a simple method to detect small numbers of nano-sized magnetic particles via the observation of magnetically captured micro-sized magnetic particles. This method is called magnetically induced self-assembly of magnetic particles [8]. The schematic image of our basic concept is described in Figure 3. First, nano-sized magnetic particles are attached on the sensing area surface via a target biomaterial. Subsequently, a solution containing micro-sized magnetic beads or columnar magnetic beads dropped onto the sensing area. Then, after applying magnetic field by permanent magnet, columnar beads are magnetically captured onto the nano-sized magnetic particles. Through the trapping of the micro-sized particles, we can detect the existence of the nano-sized particles on the sensing area by conventional optical microscope, which is a fairly inexpensive set-up.

**Figure 4:**
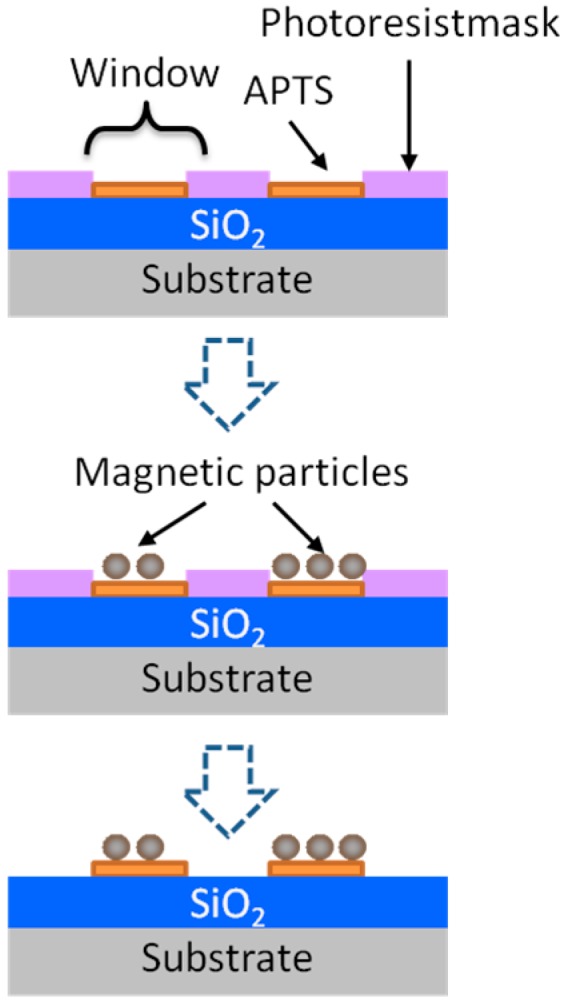
The procedure to immobilize nano-sized magnetic particles partially on the surface of thermally-oxide silicon substrate. Adapted from [8].

To test our concept, we adopted chemical binding between the nano-sized particles and the sensor surface in order to mimic the target bimolecular reaction. The procedure to immobilize nano-sized magnetic particles is summarized in Figure 4. First, a typical photolithograph technique was utilized to make windows onto the photo resist layer on the surface of thermal-oxidized silicon substrate. Then, the substrate was dipped into a solution containing aminofuntional silane [3-(2Aminoethylamino) propyltrimethoxysilane (APTS)] to make a silane-copling layer solely on the window-opened regions. Following the APTS treatment, the substrate was kept in the solution containing nano-sized magnetic particles which has a carboxyl group on the surface. Finally, partially particle-immobilized regions were obtained after a resist lift-off process. 

**Figure 5:**
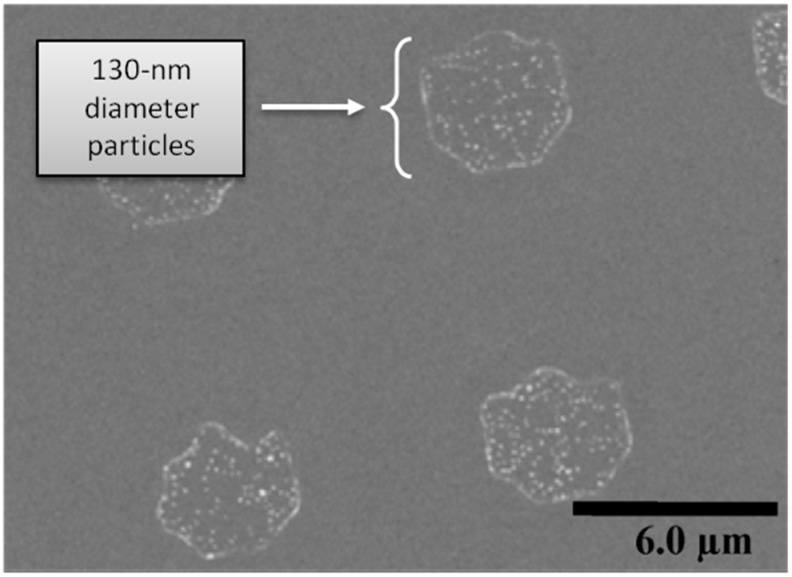
Scanning electron microscope image of immobilized 130-nm diameter magnetic particles selectively on the surface of silicon substrate. Adapted from [8].

Figure 5 shows a scanning electron microscope image of the 130-nm diameter superparamagnetic beads (Nanomag-D; magnetization 43 emu/g) on the silicon substrate using the method described above. The nano-sized particles were immobilized selectively on the surface.

**Figure 6:**
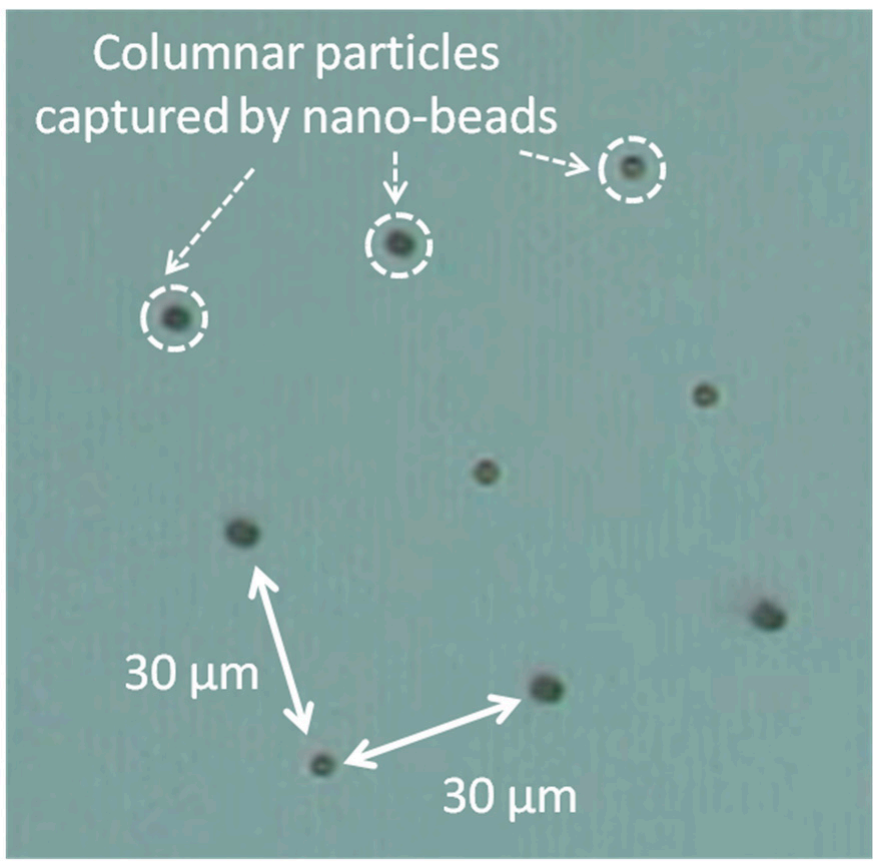
Optical microscope image of columnar particles magnetically captured by nano-sized particles immobilized on the surface of the substrate. Adapted from [8].

Figure 6 shows the 2.8-μm diameter columnar particles captured magnetically by the nano-sized particles on the substrate. It can be clearly seen that the columnar particles were only captured on the partial regions on which nano-sized magnetic particles were immobilized. Furthermore, we also successfully immobilized 200-nm diameter nano-sized particles (synthesized by Nishio *et al.* as described in [15]) on gold surface via a sulphur mediated interaction, and similarly the immobilized nano-particles were detected by columnar particle. An estimation of the minimum number of nano-sized particles was carried out; in the case of the columnar beads, this was 1.0 μm and 2.8 μm. From the experiment, at least four and 12 nano-sized particles were detected by 1.0 μm and 2.8 μm diameter columnar particles, respectively.

**Figure 7:**
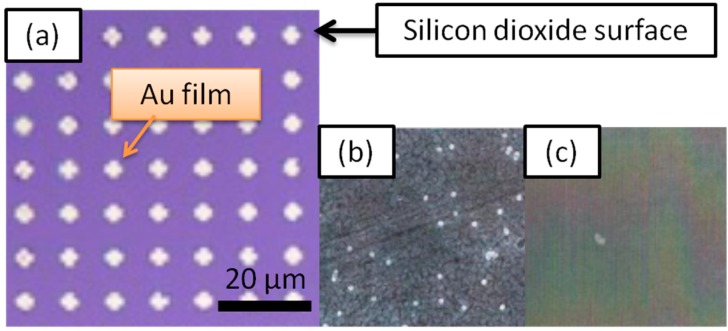
(**a**) Optical microscope image of 5 μm × 5 μm gold arrays produced on a silicon dioxide surface; (**b**) Scanning electron microscope image of 8 nm diameter thiolated superparamagnetic “target” beads immobilized via chemical affinity between the gold surface and sulfur attached to the 8 nm magnetic beads. In (**c**) SPBs were not immobilized onto SiO_2_ regions. Adapted from [9].

Further, 8-nm-diameter magnetic particles were detected by a similar method [9]. The difference was in utilizing a hand-made coil instead of a permanent magnet to apply the magnetic field. The maximum magnetic field generated by the coil was 100 Oe. The immobilization process was also different; the surfaces of the target nano-sized particles were functionalized with thiol groups. These have an affinity to gold, and the target particle can be immobilized via the reaction. Figure 7 shows the result of the immobilization of the 8-nm-diameter magnetic particles on the gold surface.

**Figure 8:**
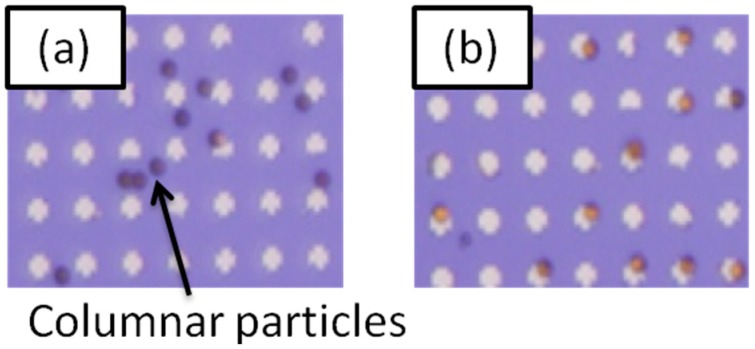
(**a**) Optical microscope image of the random distribution of columnar-beads onto gold arrays in the absence of an external magnetic field; (**b**) distribution of the columnar-beads after application of an external magnetic field of 37 Oe. Adapted from [9].

Figure 8 shows the result of 8-nm-diameter magnetic particle detection by columnar beads. The intensity of the applied magnetic field was 37 Oe. It is clearly seen that columnar beads were gathered on the gold surface after applying the field. We also investigated the time of the detection and more than 80% of columnar-beads were self-assembled onto the gold arrays within 10 s during the application of the external magnetic field. These results show our method has much potential for the development of a highly sensitive, high-speed and low cost medical diagnosis system.

### 3.2. The Amplification of the Magnetic Signal by Magnetically-Assembled Columnar Particles 

**Figure 9:**
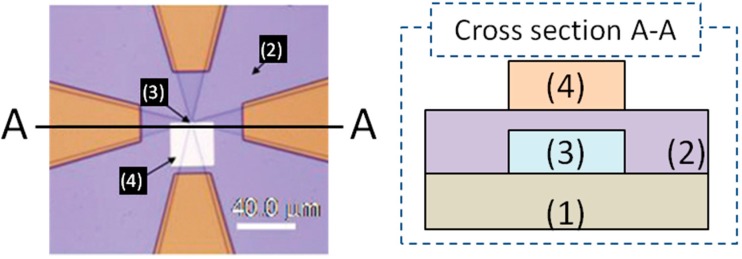
Schematic composite structure of a micro-Hall effect device. (**1**) GaAs (001) substrate; (**2**) silicon nitride insulating layers; (**3**) AlGaAs/InGaAs/GaAs Hall effect devices active layer; (**4**) Au/Ti interface layer for immobilization of functionalized nano-sized magnetic beads comparable to actual biomolecules. Adapted from [10]

We also investigated the amplification of the magnetic signal by columnar particles from the Hall-effect device measurement in DC magnetic field [10]. The configuration of the Hall sensor is described in Figure 9. Gold substrate was deposited on the center of the Hall device to immobilize the target nano-sized particles. The immobilization method was slightly different compared to our report. In this experiment, the device was dipped in cystamine dihydrochloride solution, (SCH_2_CH_2_NH_2_)_22_HCl (>98%), at room temperature for 8 h to form an amine-terminated layer on the gold surface. Carboxyl-terminated nano-sized 130-nm-diameter beads were immobilized on the gold surface as target particles.

**Figure 10:**
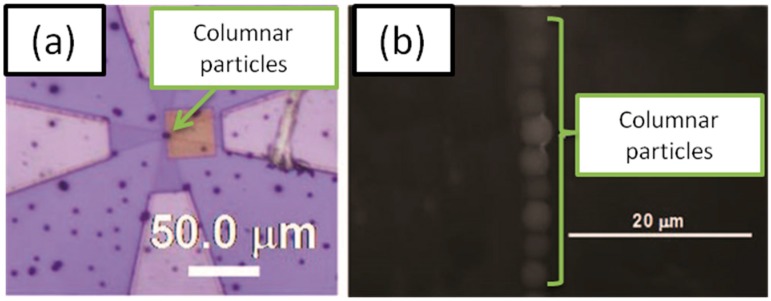
(**a**) Application of the external magnetic field gradient to form the vertical aligned self-assembled structure with respect to the surface; (**b**) The chainlike self-assembly of micro-SPBs in solidified polymer matrix observed by SEM. Adapted from [10].

Figure 10a shows the self-assembled micro-sized particles on the center of the Hall sensor on which a gold layer was deposited for immobilizing nano-sized target particles. The manipulation of columnar particle was carried out by permanent magnet to move the center of Hall sensor. To investigate the amplification effect, the columnar beads were dispersed in photocurable resin [16] and dropped on the sensor surface where they were magnetically captured by the target particle. The sensor was exposed to ultraviolet light in order to solidify the resin and keep the self-assembled configuration. Figure 10b shows the scanning electron microscope image of magnetically captured columnar particles onto the gold substrate in solidified resin. As expected, chainlike assembled columnar particles were observed. 

**Figure 11:**
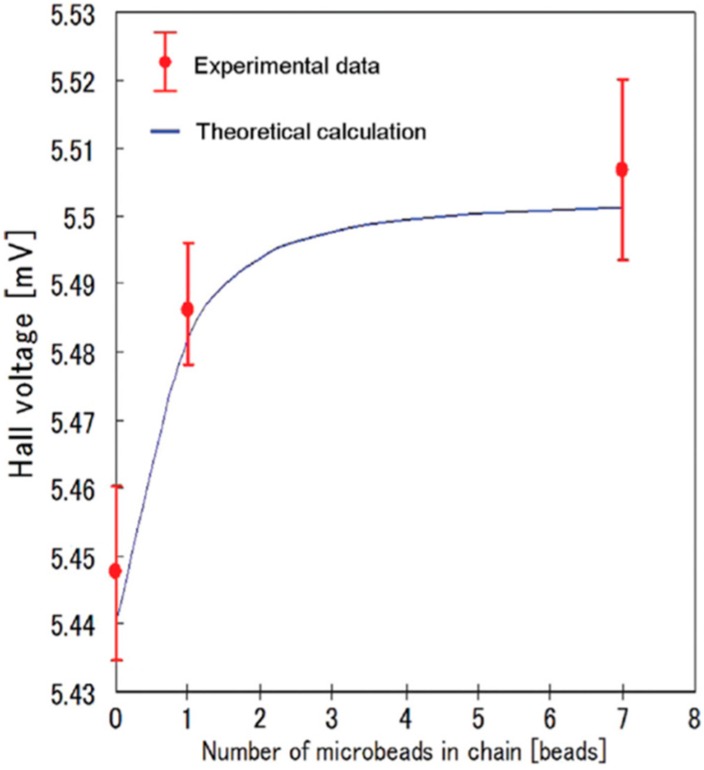
Hall voltage related to chains of self-assembled micro-SPBs bound to sensor. Data points: mean; error bars: standard deviation (*n* = 20) Adapted from [10].

Figure 11 is a graph showing the experimental and simulation output voltage of Hall sensor *vs.* the number of columnar beads with an external magnetic field of 800 Oe applied perpendicular to the sensor surface. The output was obviously increased by columnar particles and this method shows promise for detecting low concentration target nano-sized particles.

### 3.3. The Investigation of Magnetic Self-Assembly Detection in Micro Channel 

For the rapid and efficient handling of small amounts of analyte, both micro-fluidic devices and bio-sensing are powerful tools, especially for point-of-care testing. To meet such a demand, we also investigated the use of our novel detection method in micro fluidic devices. More specifically, our detection method was carried out in micro channel made from poly-dimethylsiloxane (PDMS) [11]. 

**Figure 12:**
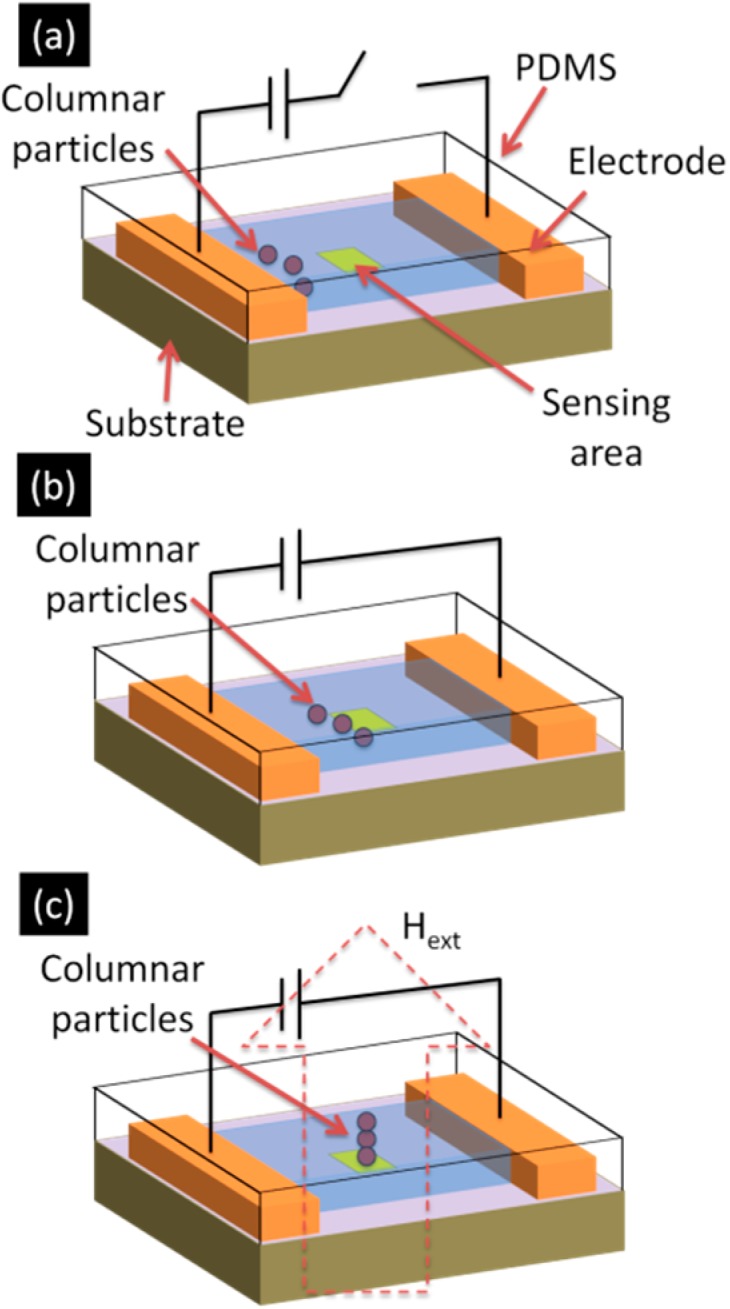
(**a**) An aqueous solution containing superparamagnetic micrometer size columnar beads was dropped into one of reservoirs; (**b**) An electric field was applied across the channels to manipulate the columnar beads; (**c**) The external magnetic field was applied to the microfluidic chip to detect the 130-nm-diameter target beads immobilized on the surface of channels. Adapted from [11].

Figure 12 is a schematic image showing how to detect nano-sized magnetic target particles in PDMS micro-channel. The unique feature of our system is that it utilizes electrostatic manipulation of columnar particles instead of an external pump. Typically, pressure-driven micro fluidic systems are often large and lack portability, which is not suitable for point-of-care testing. In our system, columnar particles, on which the carboxyl group are functionalized and have negative charges in aqueous solution, are manipulated by an electric field induced in the channel by applied voltage between the electrodes created on the edges of the channel as shown in Figure 12a. Followed by the electrostatic manipulation of columnar particles on the sensing area where target nano-sized particles were immobilized as shown in Figure 12b, magnetic self-assembly of columnar particles was induced by an external magnetic field from an electro magnet coil as shown in Figure 12c.

**Figure 13:**
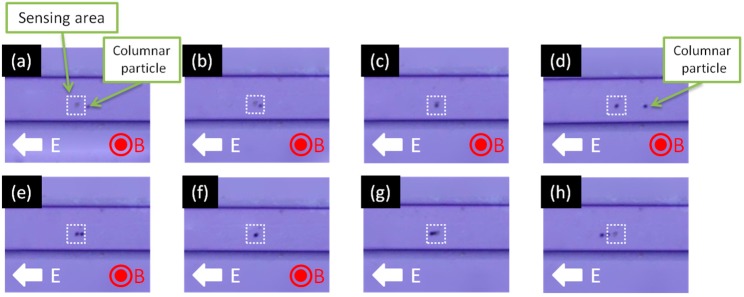
(**a**–**h**) Sequence optical images of electrostatic manipulation movement of columnar particles in micro channel filled with aqueous solution and detection of 130 nm diameter target particles via magnetically induced self-assembly of columnar particles. (**a**,**b**) Movement of a single 2.8 μm diameter magnetic particle in the microchannel; (**c**) The micrometer sized particle is magnetically captured by the immobilized target particles; (**d**,**e**) Another columnar particle is moved towards the location where the previous 2.8 μm diameter particle was captured; (**f**) Another columnar particle self-assembles onto the previous bead. (**g**,**h**) Columnar particles begin to move through the microchannel again in the absence of an external magnetic field due to the electric force still acting on the columnar particles. Adapted from [11].

Figure 13a–h show a series of images taken with an optical microscope showing 2.8-μm-diameter superparamagnetic columnar particles being manipulated by the electric field in microchannels. They are seen to self-assemble on a spot onto which the 130-nm-diameter target beads are immobilized in the application of the external magnetic field, before starting to flow through the microchannels in the absence of the external magnetic field due to the electric force acting on the columnar beads. This result indicates pump-less manipulation of columnar particles was successfully carried out and our method is achievable in microfluidic devices.

The results from these experiments demonstrate the potential for developing a highly sensitive, high-speed and low cost medical diagnosis system.

## 4. Magnetic Particles Detection by Optical-Based Method 

As well as magnetic particle detection based on magnetic sensor and self-assembly methods, optical-based detection methods were also investigated. There are two major streams in our group; one is based on rotating magnetic particles [12], and the other is based on porous silicon [13].

### 4.1. Transmittance Light-Based Biomaterial Detection in Rotating Magnetic Field 

In recent reports including our own research, magnetic particles’ labeling detection yielded reasonably high sensitivity. However, such detection methods still require “solid state” based biosensors which are fabricated by highly-technical equipment resulting in increasing costs. A partial functionalized surface is also required on the devices. A new highly sensitive, inexpensive and simple method is in demand. To overcome such difficulties, we developed a detection system based on transmitted light changes which is dependent on the length of the nano-sized magnetic particles’ chain in the rotating magnetic field [12].

**Figure 14:**
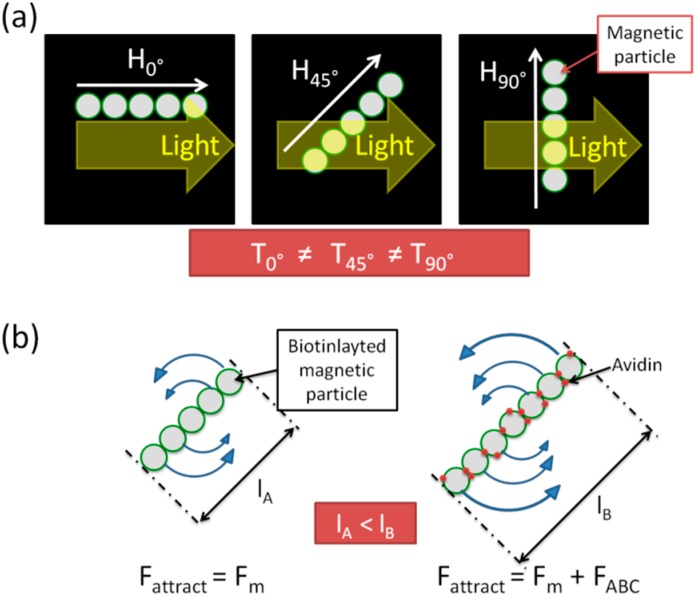
(**a**) schematic image of the chain-shape nano-sized particles by rotating magnetic field. Transmitting light has a dependence of the angle between the direction of the chain and the path of the light; (**b**) length dependence of the biotinlayted nano-sized magnetic particles in rotating magnetic field with and without avidin. Because avidin interconnects biotin, the force between the particles is the sum of the magnetic interaction and the biological conjugation force via avidin–biotin interaction resulting in the increase of the chain length. Adapted from [12].

The concept of this method is described in Figure 14. This detection method is based on Rayleigh-Gans scattering by the particles’ chain. In the presence of a magnetic field, each nano-sized magnetic particle is magnetized, magnetically attracted to each other, and finally form a chainlike shape. The scattering is affected by the chain length and the angle between the direction of the chain and the light path as shown in Figure 14a. Furthermore, the chain length is determined by N ≈ (1/Mn)^0.5^ where N is the number of nano-sized particles in the formed chain in rotating magnetic field and Mn indicates the Mason number. This is defined as follows;

(1) $$\text{Mn}=\frac{{\text{F}}_{\text{H}}}{{\text{F}}_{\text{m}}}\approx \frac{16\text{ηω}}{{\text{μ}}_{0}{\text{χ}}^{2}{\text{H}}^{2}}$$

where F_H_ is the hydrodynamic force, F_m_ is the magnetic dipolar force, η is the viscosity of surrounding fluid, ω is the angular velocity of the field, μ_0_ is the permeability of free space, and χ is the magnetic susceptibility of nano-sized particles. In the case of introducing avidin into the biotinylated nano-sized particles’ solution, the biological conjugation force is added to the interaction force of magnetic particles and increases the chain length, finally causing changes in the intensity of the transmitted light.

**Figure 15:**
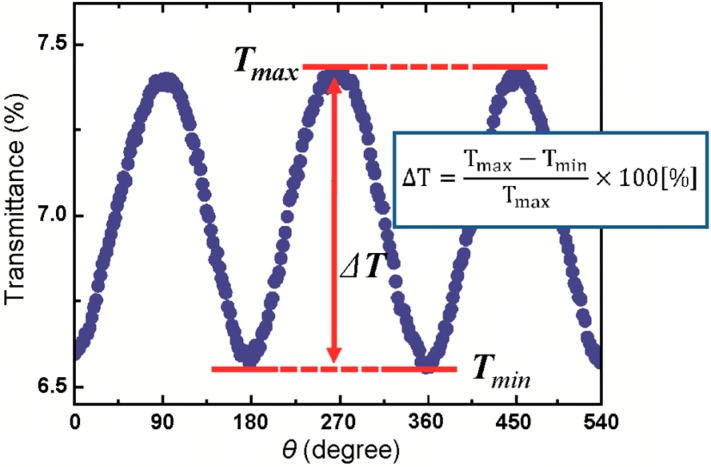
Time dependence of the transmitted light through the particle solution in a rotating magnetic field with frequency 0.1 Hz. Adapted from [12].

Figure 15 shows the time dependence of the transmitted light through the particle solution in a rotating magnetic field with a frequency of 0.1 Hz. We can define a magnetic transmittance (MT) ratio (%), (T_max_ − T_min_)/T_max_ × 100, where T_max_ and T_min_ are the light intensity transmittance when the directions of the chain to the light path are parallel and perpendicular, respectively. 

**Figure 16:**
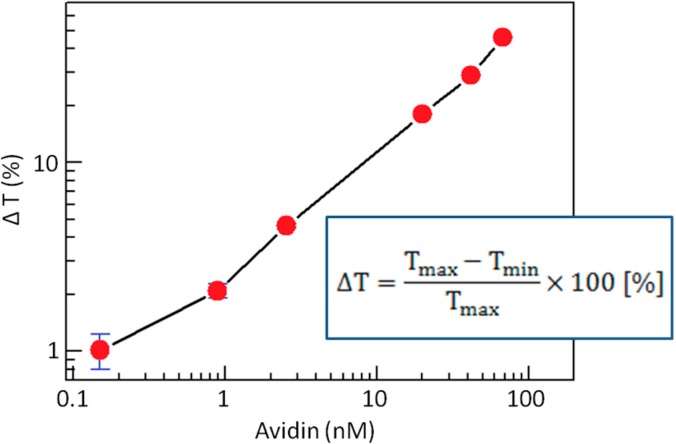
A graph of transmitted light ration *vs.* the concentration of avidin added into biotinylated 200 nm-diameter particles’ solution. Adapted from [12].

Figure 16 shows the results for the detection of avidin using 200 nm diameter biotinylated nano-particles. The biotinylated particles were covered with 6 × 10^6^ biotin groups/beads. The solution was irradiated with collimated white light and the 785 nm wavelength of transmitted light was monitored. The rotation frequency and external magnetic fields were 0.1 Hz and 0.95 kA·m^−1^, respectively. This procedure enabled the detection of avidin to a sensitivity of ~100 pM (6.7 ng/mL) over a dynamic range of at least three orders of magnitude. Notably, the rotating chains acted as both biomolecule probes and micromagnetic mixers, enabling detection of biomolecular recognition in less than 30 s.

### 4.2. Porous Silicon-Based Detection via the Reflectance Intensity Changes by Tager Nano-Sized Particle

For the realization of a highly sensitive, inexpensive and easy-to-use biomaterial detection system, a porous silicon (PSi) based platform also has great potential. Normally PSi is fabricated by electrochemical anodization and can be accurately tuned by controlling the geometric orientation, size, and thickness of the pores in its surface. Moreover, PSi has a large surface area which is suitable for bio-material conjugation on the surface. We demonstrate the optical detection of low densities of sub-300 nm SPBs on multi-layer-PSi in less than 60 s.

**Figure 17:**
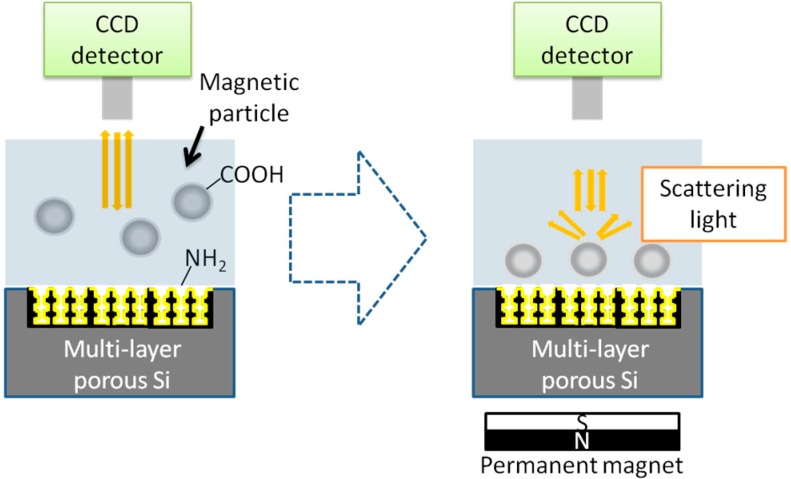
The schematic image of the nano-sized target particles detection based on multi-layer-PSi. Adapted from [13].

Figure 17 is a schematic image of our biosensing protocol based on multi-layer-PSi. Multi-layered-PSi was obtained in a repeated electro chemical anodization where a repeating cycle of a high current was applied for a short time and a small current was applied for a long time, repeatedly. Because of such periodicity, the reflection light of multi-layer-PSi has a specific peak according to Bragg’s Law. 

**Figure 18:**
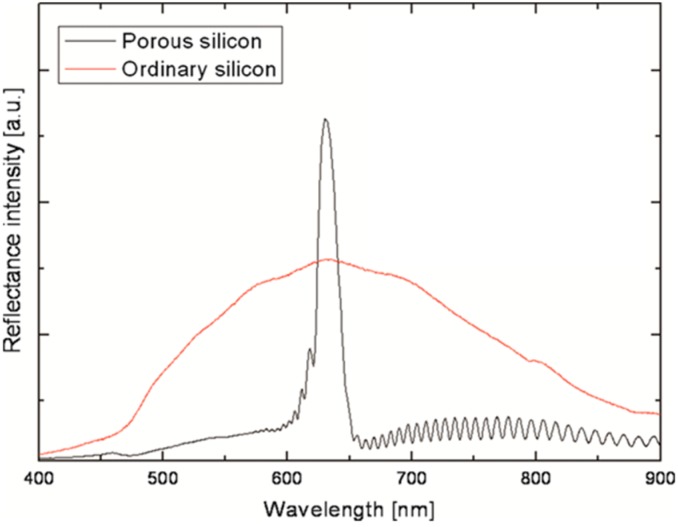
The comparison of the reflected intensity light between ordinary silicon and multi-layer-Psi. Adapted from [13].

Figure 18 shows the intensity of the reflectance of white light as a function of wavelength. This multi-layer-PSi was prepared by using a periodic square-wave current that alternated between low (90 s at 5 mA·cm^−2^) and high (3 s at 50 mA·cm^−2^) current densities, repeated 30 times. Compared to ordinary silicon reflection, our fabricated sample has a strong peak of around 630 nm.

**Figure 19:**
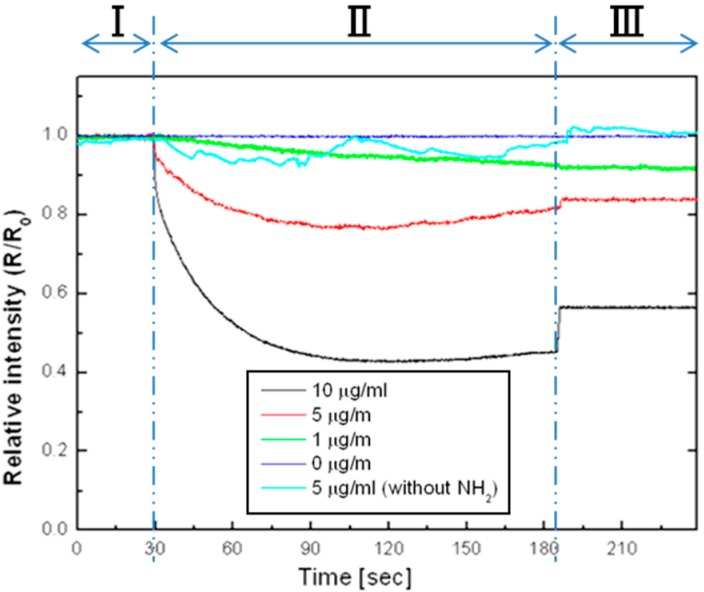
The reflective intensity from the multi-layer-PSi Region I: Absence of a magnetic field (H = 0). Region II: Application of a magnetic field (H = 500 Oe) Region III: After rinsing with PBS solution without a magnetic field (H = 0). Adapted from [13].

Figure 19 shows the degree of change in the intensity of reflected light at 630 nm as a function of time for several concentrations of target particles’ solution. The experimental procedure is as below. First, the multi-layer-PSi surface was thermally oxidized and treated with APTS solution to functionalize amino group and was subsequently covered with phosphate buffer solution. Colloidal solution consisting of carboxyl-terminated magnetic particle (Nanomag-D, 250 nm diameter, Micromod Inc., Rostock, Germany), which was already activated by 1-ethyl-3-(3-dimethylaminopropyl) carbodiimide (EDC), was dropped onto the surface of multi-layer-PSi. All reflected light intensity in the graph was normalized by each intensity when the solution containing target nano-sized particles dropped onto the surface of multi-layer-PSi.

Region I indicates the signal before applying magnetic field. Because of the well-dispersed 250 nm diameter target particles, there are no specific intensity changes. Region II refers to the application of a magnetic field gradient by a NdFeB magnet underneath the sample to collect magnetic particles on the surface of the substrate. Each reflected light intensity obtained in each concentration of magnetic particles dramatically started to reduce and was saturated in less than 60 s. Moreover, no amino-modified multi-layer-PSi (light blue) was able to confirm that the intensity change was obtained due to the surface reaction between the target particles and multi-layer-PSi. These results show the light scattering method is compatible with use for rapid, real-time, point-of-care medical diagnostics.

## 5. Recent Work with Industrial Products

In terms of more practical and industrial applications, we are now developing a smart-phone-based system for detecting nano-sized target magnetic particles. The technique of “magnetic washing”, which is used for magnetic removal of unspecific-binded nano-sized particles on the substrate, is being investigated. We would like to briefly summarize recent works as below.

### 5.1. Smartphone-Based Photonic Biosensing Protocol 

In some developing countries, the medical infrastructure is not well established and diseases such as tuberculosis and malaria cause serious social disruption. Hence, there is demand for rapid and easy-of-use medical diagnosis to resolve this situation. 

Intriguingly, the number of smartphone users in developing countries is increasing rapidly, and the possibility of conducting medical diagnosis based on smartphones would offer an innovative method for treatment of diseases.

**Figure 20:**
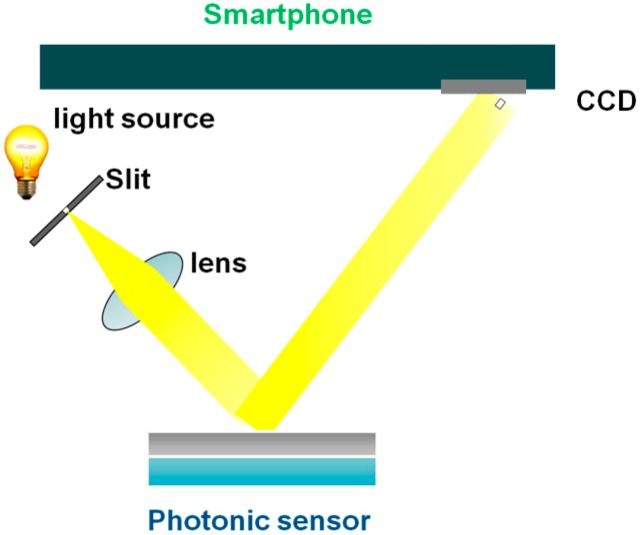
Our concept of a smartphone-based medical diagnosis system

Figure 20 shows the schematic image of our smartphone-based system for medical diagnostics. It consists of incident light, lens, photonic sensor and smartphone. We are now developing dedicated software to monitor changes of the output signal from the photonic sensor. We will report our findings in the near future.

### 5.2. Magnetic Removal of Unspecific-Binded Nano-Sized Particles-“Magnetic Washing”

**Figure 21:**
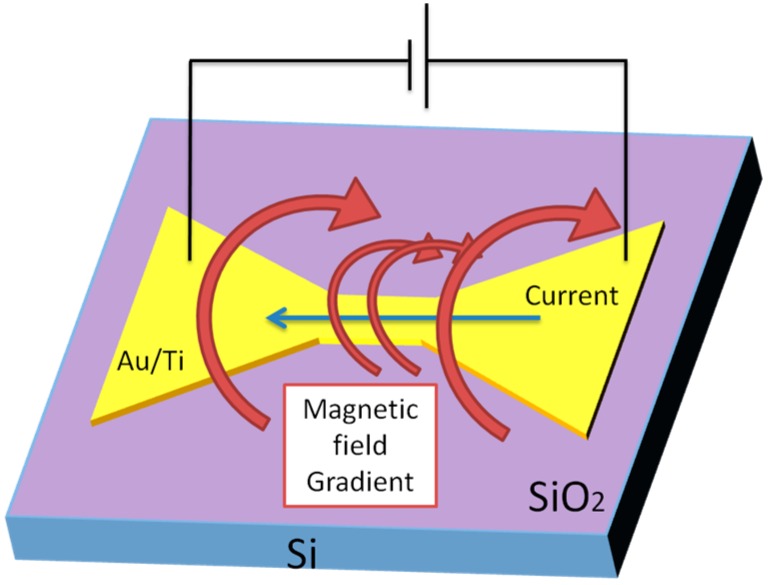
The basic concept of “magnetic washing” via magnetic field gradient, induced by an applied current thorough a gold layer. Adapted from [14].

We are also developing the technique of “magnetic washing” for magnetically removing nonspecifically-bonded nano-sized particles on the surfaces of biosensors. Our approach is based on a magnetic field gradient induced by a current passed through gold micro-lines integrated with the substrate [14] as shown schematically in Figure 21. The magnitude of the magnetic field gradient is controlled by varying the dimensions of the gold pattern microlines as described in a report on Hall effect biosensors.

## 6. Conclusions

We have reviewed our recent results of detecting nano-sized magnetic particles. 

First, we discussed detection using Hall effect magnetic sensors. The detection was carried out based on a lock-in amplifier to monitor changes in the magnetization of target magnetic particles with and without sufficient magnetic field. From our results, the detection of a 2.8 μm diameter magnetic bead was successful. We also succeeded in the detection of DNA molecules labeled with 200 nm diameter magnetic beads using the same detection system.

We summarized the magnetically-induced self-assembly beads phenomena for detecting nano-sized magnetic particles immobilized on the surface of sensors. In this section, the procedure behind detecting such small target particles was clearly described, as were the results for the detection of 130-nm-diameter particles with a carboxyl surface group, and 8-nm-daimeter-biotinlated particles via magnetic capture of columnar beads using optical microscopy. The amplification of the magnetic signal due to the captured columnar particles was investigated by Hall magnetic sensor, and the amplified signal was successfully obtained. Furthermore, the detection method was investigated in PDMS-microchannel for a practical application, and we confirmed that our method shows promise for the development of a pump-free, highly sensitive, and low cost medical diagnosis system. 

Detections based on optical methods were also reported. The method of measuring the intensity of transmitted light through the nano-sized particles solution in rotating magnetic fields confirmed successful detection with a wide range of concentrations of avidin from sub-nanomolar to several-tens nanomorlar in 30 s. We demonstrated the detection of porous silicon-based nano-sized magnetic particles shows good potential for rapid, real-time, point-of-care medical diagnostics.

Finally, our most recent experiments were briefly summarized. Two topics were reported; one is about the development of a smartphone-based photonic sensor detection protocol, and the other concerns magnetically removing unspecific-binded nano-sized particles. The details of each topic will be reported in the future. 

## References

[1] Van Reenen A., de Jong A.M., den Toonder J.M. J., Prins M.W. J. (2014). Integrated lab-on-chip biosensing systems based on magnetic particle actuation—A comprehensive review. Lab Chip.

[2] Pankhurst Q., Thanh N., Jones S., Dobson J. (2009). Progress in applications of magnetic nanoparticles in biomedicine. J. Phys. D Appl. Phys..

[3] Sandhu A., Sanbonsugi H., Shibasaki I., Abe M., Handa H. (2004). High sensitivity InSb ultra-thin film micro-Hall sensors for bioscreening applications. Jpn. J. Appl. Phys. Part 2 Lett..

[4] Primadani Z., Osawa H., Sandhu A. (2007). High temperature scanning Hall probe microscopy using AlGaNGaN two dimensional electron gas micro-Hall probes. J. Appl. Phys..

[5] Ohashi T., Osawa H., Sandhu A. (2008). Contact mode scanning Hall probe microscopy. IEEE Trans. Magn..

[6] Bando M., Ohashi T., Dede M., Akram R., Oral A., Park S.Y., Shibasaki I., Handa H., Sandhu A. (2009). High sensitivity and multifunctional micro-Hall sensors fabricated using InAlSb/InAsSb/InAlSb heterostructures. J. Appl. Phys..

[7] Togawa K., Sanbonsugi H., Sandhu A., Abe M., Narimatsu H., Nishio K., Handa H. (2006). Detection of magnetically labeled DNA using pseudomorphic AlGaAs/InGaAs/GaAs heterostructure micro-Hall biosensors. J. Appl. Phys..

[8] Morimoto Y., Abe M., Hatakayama M., Handa H., Sandhu A. (2009). Detection of magnetic nanobeads by self-assembly of superparamagnetic microbeads for biosensing. IEEE Trans. Magn..

[9] Morimoto Y., Takamura T., Park S.Y., Sakamoto S., Kawata S., Handa H., Sandhu A. (2010). Detection of 8 nm diameter superparamagnetic beads by magnetically-induced manipulation of micrometer-sized magnetic beads: A novel protocol for magnetically-labeled biosensing. Jpn. J. Appl. Phys..

[10] Morimoto Y., Takamura T., Ishikawa R., Ko P.J., Sandhu A. (2011). Amplification of direct current magnetic responses of magnetic nanobeads due to induced self-assembly of magnetic microbeads. J. Appl. Phys..

[11] Morimoto Y., Takamura T., Sandhu A. (2010). Compact electromagnetically operated microfluidic system for detection of sub-200-nm magnetic labels for biosensing without external pumps. J. Appl. Phys..

[12] Park S.Y., Handa H., Sandhu A. (2010). Magneto-optical biosensing platform based on light scattering from self-assembled chains of functionalized rotating magnetic beads. Nano Lett..

[13] Ko P.J., Ishikawa R., Takamura T., Morimoto Y., Cho B., Sohn H., Sandhu A. (2011). Porous-Silicon Photonic-Crystal Platform for the Rapid Detection of Nano-Sized Superparamagnetic Beads for Biosensing Applications. Nanosci. Nanotechnol. Lett..

[14] Kumagai Y., Togawa K., Sakamoto S., Abe M., Handa H., Sandhu A. (2006). Hall biosensor with integrated current microstrips for control of magnetic beads. IEEE Trans. Magn..

[15] Nishio K., Masaike Y., Ikeda M., Narimatsu H., Gokon N., Tsubouchi S., Hatakeyama M., Sakamoto S., Hanyu N., Sandhu A. (2008). Development of novel magnetic nano-carriers for high-performance affinity purification. Colloids Surf. B Biointerfaces.

[16] Lee H., Kim J., Kim H., Kim J., Kwon S. (2010). Colour-barcoded magnetic microparticles for multiplexed bioassays. Nat. Mater..

